# The Caryopsis of Red-Grained Rice Has Enhanced Resistance to Fungal Attack

**DOI:** 10.3390/jof4020071

**Published:** 2018-06-14

**Authors:** Alberto Gianinetti, Franca Finocchiaro, Fabio Maisenti, Dailly Kouongni Satsap, Caterina Morcia, Roberta Ghizzoni, Valeria Terzi

**Affiliations:** Council for Agricultural Research and Economics—Research Centre for Genomics and Bioinformatics, via S. Protaso 302, 29017 Fiorenzuola d’Arda (PC), Italy; franca.finocchiaro@crea.gov.it (F.F.); fabio.maisenti@studenti.unipr.it (F.M.); satsapdailly@yahoo.fr (D.K.S.); caterina.morcia@crea.gov.it (C.M.); roberta.ghizzoni@crea.gov.it (R.G.); valeria.terzi@crea.gov.it (V.T.)

**Keywords:** seed fungal infection, germination, seedling growth, pigmented rice, red rice, proanthocyanidins, grain mycotoxins

## Abstract

Seed persistence in the soil is threatened by microorganisms, but the seed coat helps protect the seed from them. Although modern rice (*Oryza sativa* L.) cultivars have a whitish caryopsis, some varieties have a red caryopsis coat, a trait typical of wild *Oryza* species. The red colour is due to the oxidation of proanthocyanidins, a class of flavonoids that is found in the outer layers of the seed in many species. We aimed to assess whether these natural compounds (proanthocyanidins and proanthocyanidin-derived pigment) have some protective effect against microbial attacks. Dehulled caryopses of white-grained and red-grained rice genotypes were employed to assay fungal infection. Specifically, three white-grained rice cultivars (Perla, Augusto, and Koral) and three red-grained rice varieties (Perla Rosso, Augusto Rosso, and Koral Rosso) were used. In a first test, the caryopses were infected with *Epicoccum nigrum* at 10 °C, and seedling growth was then assessed at 30 °C. In a second test, the degree of infection by the mycotoxigenic fungus *Fusarium sporotrichioides* was assayed by measuring the accumulation of T-2/HT-2 toxins in the caryopses. Infection was performed at 10 °C to prevent rice germination while allowing fungal growth. In both the tests, red caryopses showed reduced, or delayed, infection with respect to white ones. One black-grained cultivar (Venere) was assayed for the accumulation of T-2/HT-2 toxins as well, with results corresponding to those of the red-grained rice varieties. We argue that the red pigment accumulating in the caryopsis coat, and/or the proanthocyanidins associated with it, provides a protective barrier against challenging microorganisms.

## 1. Introduction

Pigmented rices have a kernel (dehulled caryopsis) with either a black or red-brick colour and are characterized by the presence of high concentrations of flavonoids in the outer layers of the caryopsis [1,2]. Specifically, red-grained rice accumulates proanthocyanidins (PAs) [3], whereas black-grained rice accumulates anthocyanins [2].

Seed germination and seedling vigour are negatively affected by the growth of plant pathogenic micro-organisms, particularly fungi [4,5]. Fungal growth can therefore negatively impact the establishment of a good standing crop [5,6]. The rice dispersal unit is a spikelet, that is, a kernel tightly covered by the hull, which is a first, mechanical barrier protecting the intact grain [7]. In addition to the hull, which is a dead tissue, chemical defences like phenolics are frequently associated with the seed, or caryopsis, coats, and PAs play an important role in the defence against pathogens and predators (fungi, bacteria, and insects) specifically in the seed [8,9]. Besides, even the mechanical resistance of the caryopsis coat could be enforced, as PAs polymerize to large, poorly soluble phlobaphenes [3,10]. In barley, a study of mutants showed that the presence of PAs is important for resistance toward pathogenic fungal species, since penetration of *Fusaria* into the caryopsis is increased when flavonoids (PAs) production is suppressed [10].

Several species of fungi of the *Fusarium* genus can produce toxic secondary metabolites, referred to as mycotoxins, which accumulate in the cereal grain [11,12,13]. This causes a reduction in grain quality and constitutes an important food safety issue [12]. Among the measures that can be applied to control mycotoxins, the adoption of genetically resistant plant varieties and appropriate agronomic practices are primary strategies able to prevent or reduce the fungal development and production of toxic metabolites in the field [12]. As a broad-spectrum genetic plant defence mechanism against pathogens, accumulation of phenolic compounds has been shown to inhibit the in vitro growth and reproduction of a wide array of fungal genera and can help in reducing *Fusarium* trichothecene mycotoxin accumulation in cereal grains [11].

Moreover, sprouted rice can be used for human consumption: germinated brown rice has gained significant attention during the last decade as a tool for enhancing eating quality because of its potential health-promoting functions [14]. Pigmented rices, with either a red or black caryopsis, have been employed for this specific use too [14]. Of course, microbial contamination can be an issue for this product [14], and growth of mycotoxigenic fungi must be obviously prevented. It would therefore be interesting to ascertain whether the pigmented caryopsis has some advantage in this respect. Although our approach was not directly aimed to this latter scope, our study may be of relevance for this application too, since microbial growth during germination can render sprouted rice unsuitable as food material, particularly if germination is extended [14].

In this work, we used dehulled caryopses of white-grained and red-grained rice genotypes to study whether kernel pigmentation can affect fungal infection development. In a first test, the caryopses were infected with *Epicoccum nigrum* to study its effects on seed germination and seedling vigour. In a second test, the amount of T-2/HT-2 toxins (type A trichothecenes) that accumulated in the caryopses following infection by *Fusarium sporotrichioides* was assayed. An effect of the caryopsis pigmentation was evident in both cases.

Neither *E. nigrum* nor *F. sporotrichioides* are major rice pathogens. However, *E. nigrum*, a world-wide spread saprophytic fungus [15], is commonly found on rice spikelets, where it can cause a pink to red discoloration of rice kernels, known as red blotch of grains [5,16]. On the other hand, *F. sporotrichioides* is widespread on plants and in soil across temperate regions and is known to infect grains of rice and other cereals with substantial production of type A trichothecenes [17]. These two species were used in our experiments because of their ability to grow at a low temperature (10 °C), at which rice germination cannot occur.

## 2. Materials and Methods

### 2.1. Rice Genotypes and Kernel Preparation

Kernels of three white-grained rice cultivars (Perla, Augusto, and Koral), three red-grained rice varieties (Perla Rosso, Augusto Rosso, and Koral Rosso; the Italian word “rosso” means “red”), and one black-grained cultivar (Venere) were harvested in the greenhouse at Fiorenzuola d’Arda (Italy) and stored at 5 °C for about one year to remove dormancy.

Each red-grained variety originated from a single red-grained plant found in a plot sown with the white-grained cultivar from which the variety has taken its name, and it shows close phenotypic similarity to the corresponding cultivar but for the red kernel. Almost every cultivated rice (*Oryza sativa* L.) genotype carries a 14 bp deletion at the *Rc* locus responsible for the accumulation of PAs in wild *Oryza* species (and in weedy rices), and this mutation is responsible of the whitish colour of the caryopsis in crop rice [18]. Whereas Perla Rosso derived from the white-grained cultivar Perla by a reverse mutation at the *Rc* locus [19], in the two other white/red grained genotype pairs, the genetic relationship between the parent cultivar and its red-grained variant is unknown. Although the genetic background is, therefore, not necessarily identical for the two genotypes of each pair (a red variety could eventually be a hybrid progeny displaying phenotypic convergence toward the white parent but carrying the red grain trait as inherited from a weedy parent), pairwise comparison of white and red grained genotypes with close phenotype (like corresponding grain shape and size) was carried out in order to minimize possible interferences.

Rice spikelets were manually dehulled before each experiment. Kernels (dehulled caryopses) were singularly checked with a stereoscopic microscope to remove damaged caryopses. In fact, a crack in the caryopsis coat, produced sometimes during dehulling, represents an open door for microorganisms to access the interior of the kernel, thus spoiling experimental results.

### 2.2. Effect of Red Rice Pigment Extract on Fungal Growth

A preliminary experiment was done to test whether red rice pigment extract (containing polyphenols, mainly PA-associated compounds [3]) has an inhibitory action on fungal growth. To this aim, 30 g of dehulled kernels was defatted with 25 mL of hexane for two hours. Then, the kernel sample was extracted for two hours with 25 mL of 70:30 (*v*/*v*) acetone/water, which allows extraction of PAs and PA-associated compounds [3]. The extract was filtered and brought to dryness with a vacuum rotary evaporator (Heidolph Instrument, Kelheim, Germany) at 38 °C. The dried residue (2.6 g) was re-dissolved in 15 mL of 5% (*w*/*v*) polyethylene glycol (PEG) 8000 solution in water. As the red pigment and polymeric PAs are poorly soluble in water [3], PEG was used to dissolve these compounds in an aqueous solution [20].

The pigment extract (in 5% PEG solution) was included in Potato Dextrose Agar (PDA) medium (15 g/L agar) at concentrations of 0.5%, 1%, 2%, 4%, and 8% (*v*/*v*). Final PEG concentration in the medium was ≤0.4%. These enriched media were used for the growth of *Fusarium graminearum*, *Fusarium subglutinans* and *Fusarium verticillioides* (supplied by the Catholic University of Piacenza, Italy). Plates (60 mm diameter) with the enriched media, and control plates with either basic PDA medium or PDA including corresponding concentrations of 5% PEG solution (without pigment extract), were inoculated in the centre with a 5-mm round plug from PDA plates with actively growing mycelium and incubated at 22 °C under fluorescent light (12 h photoperiod) for six days. Mycelial growth was evaluated as the mean colony diameter measured in two perpendicular directions [21]. The effect of the pigment extract on fungal growth was expressed as inhibition percentage (I), calculated according to the formula I = [(C − T)/C] × 100, where C is the control plate colony diameter, and T is the treated plate colony diameter [21]. The standard error (SE) for the percentage of inhibition (I) was calculated by error propagation (assuming that the measurements of C and T are independent). Duplicate plates were used for every tested condition.

### 2.3. Fungi Used for Infection Tests

*Fusarium sporotrichioides* strain 692 was provided by the Istituto di Scienze delle Produzioni Alimentari (ISPA-CNR, Bari, Italy). A strain of *Epicoccum nigrum* Link, found to grow at 10 °C on a rice kernel incubated on water agar, was isolated from a hyphal tip and transferred to a sterile PDA plate. The taxonomic identity of *E. nigrum* was established on the basis of the morphology and colour of conidia and of the macroscopic colony, which abundantly released a purple exudate into the medium. Both fungi were maintained on PDA. Though *E. nigrum* is presently considered as a single variable species, it could actually include separate species [15].

### 2.4. Effect of E. nigrum on Rice Germination and Seedling Growth

Dehulled caryopses were disinfected with a solution containing 1:1 (*v/v*) commercial bleach (2.0% NaOCl) and 0.6 M NaH_2_PO_4_ (final concentration: 0.3 M NaH_2_PO_4_; final pH: around 6.4), for 10 min, then rinsed with distilled water [22,23]. Preliminary trials showed that treating red-grained caryopses with bleach or bleach/NaH_2_PO_4_ caused visible dissolution of the red pigment if prolonged for 15 min or more.

Twenty kernels were arranged along a circle at about 1 cm from the border of a Petri dish (60 mm diameter) containing water agar (1.5%) medium. Kernels were disposed with their main axis radial to the plate centre. For fungal infection, a 10-mm mycelial disc was placed at the centre of the plate. Controls without mycelial disc were included.

Plates were incubated at 10 °C until the mycelium reached all the caryopses and started to grow on them. Then, the plates were further incubated at 30 °C for three days. Germinated seeds, shoot length and rootlet length were recorded. A vigour index was then calculated as [24]:

Vigour Index = Germination (%) × Mean seedling length (rootlet + shoot, mm)

Two to four replications were employed for every tested condition. The whole experiment was repeated twice.

### 2.5. Accumulation of T-2/HT-2 from F. sporotrichioides in Rice Kernels

In this test, kernels were incubated on water agar plates with or without inoculation with a conidial suspension of *F. sporotrichioides*. The fungus was grown on PDA plates (90 mm diameter) at 25 °C for about two weeks with a 12 h photoperiod to stimulate sporulation of the mycelium covering the plate. Then, 2 mL of sterile distilled water was placed onto the solid medium surface and the plate was gently swirled to disperse conidia into the fluid. The conidial suspensions from 3 to 5 plates were collected into a plastic tube, and then the conidia concentration was determined by means of a Bürker chamber, and the spores were counted with a microscope (BX51TF, Olympus Corporation, Tokyo, Japan). The conidial suspension was eventually diluted to adjust the final volume necessary to inoculate the plates for infecting the caryopses.

Plates (60 mm diameter) with water agar (15 g/L) medium were inoculated with 500 µL of *F. sporotrichioides* conidial suspension and left open under sterile hood to dry/absorb the added fluid. The inoculum density was always ≥8.6 × 10^6^ conidia/mL, corresponding to ≥4.3 × 10^6^ conidia per plate, to ensure a quick and simultaneous infection of caryopses. Preliminary work showed that this conidial concentration provided an excess of inoculum capable of ensuring maximal reduction of the time required for caryopses infection under our experimental conditions.

Seven dehulled rice caryopses were placed onto the medium surface. The small number of kernels was chosen to minimize the probability of having a damaged caryopsis in the plate. In fact, preliminary tests revealed that, occasionally, the kernels from some plates showed an anomalously high level of mycotoxins. The presence of these anomalous samples was probably due to at least one damaged caryopsis having escaped removal during sample preparation. Reducing the number of kernels per plate was found to reduce the frequency of anomalous samples and improve the effectiveness of the test. Removing damaged caryopses (at least, those that could be detected) and using a small number of kernels for each plate were found to strongly reduce the frequency of anomalous plates (only a very few plates appeared as outliers, and their effect on sample means were therefore limited, though they caused an increase of the variability between replicates) and were therefore systematically adopted for all the described experiments. Controls without conidial inoculum were included.

Plates were incubated at 10 °C. All the kernels from a plate were collected to provide an analytical sample. Two to four replications were used for each tested condition. Sampling of all the caryopses from independent plates was performed daily after 3 to 6 days for cultivar Perla and Perla Rosso to establish the best timing for discrimination. This experiment was repeated twice. The best sampling time was then chosen to test the other genotypes. Samples were stored at −80 °C before mycotoxin analysis.

### 2.6. Mycotoxin Quantification

Rice kernel samples collected during the experiment were ground with liquid nitrogen. A volume of 70:30 methanol/water (*v*/*v*) equal to five times the weight of the sample was added. Samples were shaken for 10 min and then centrifuged at 3000× *g* for 15 min. The filtrate was collected and diluted 1:1 with distilled water.

Competitive direct immunoassay (ELISA) was used for mycotoxin quantification [25]. The amount of T-2 and HT-2 toxins (as a sum of toxins) was determined using the kit Veratox^®^ for T-2/HT-2 assay (product code 8230, Neogen Corporation, Lansing, MI, USA). The photometric reading was done at 630 nm, according to the manufacturer’s instructions. The range of quantification of toxins for this assay is 25–250 µg/kg. The sample extracts exceeding 250 µg/kg were further diluted and reanalyzed, and the dilution factor was used to calculate the actual concentration. Values of mycotoxin concentration below 25 µg/kg were reported, but, according to the manufacturer’s instructions, they cannot be considered significantly different from zero (thus that 25 µg/kg represents the lower limit of detection, LOD).

### 2.7. Statistical Analysis

Within each white/red genotype pair, the effects of grain type, fungal treatment (presence/absence of *E. nigrum* inoculum) and their interactions on seedling growth (three measure sets were considered: rootlet length, shoot length, and their sum) were evaluated with ANOVA (analysis of variance, which was performed by using the General Linear Model procedure of Systat 12 software, SPSS Inc., Chicago, IL, USA). Tukey’s test was used to evaluate the significance of the differences within each measure set for each white/red genotype pair.

For germination percentage, mean seedling length, and vigour index, the Tukey’s test was used to evaluate the significance of the differences between cell (grain type x fungal treatment) means once nested ANOVA had shown the significance of the main effects and of their interaction. Both grain type (red/white) and fungal treatment were considered fixed factors. Genotype was considered as an additional random factor nested within grain type to account for the variability across genotypes, and with the purpose of generalizing the findings. Plates were the independent experimental units. Because of unbalanced replications, which, in the presence of random factors additional to the error requires a maximum likelihood estimation method, like restricted maximum likelihood (REML, which produces unbiased estimates of variances) [26], ANOVA was performed by using the procedure for linear mixed models of Systat 12 software. In addition, as germination percentages showed a significant inhomogeneity of variances (according to Levene’s test), with higher germination means showing lower errors and therefore with error variances correlated with germination means and closer germination means having more correlated errors, a first-order autoregressive covariance structure of the error term, which assumes that the errors are neither independent nor constant [26], was specified (for germination data only). No improvement of the significance levels obtained from ANOVA and Tukey’s test was observed if germination data were analysed after transformation to the arcsine of the square root of the proportion.

For mycotoxin values, when a single sampling day was available, data were subjected to two-ways ANOVA, with both grain type and fungal treatment as fixed factors. For data analysed across two days, three-ways ANOVA was used, and sampling day was dealt with as a categorical factor. To analyze data obtained over more than two days, the square root transformation was adopted because ANOVA factors must show additivity [27], which, in the case of time, means linearity of outcomes through time, an assumption that did not hold for mycotoxin accumulation. Time was then used as a covariate in ANOVA to evaluate the effect of grain type during fungal treatment. Plates were the independent experimental units. ANOVA was performed by using the General Linear Model procedure of Systat 12 software.

## 3. Results and Discussion

The outer structures of plants dispersal units are the first defence against micro-organisms threatening seed integrity and survival. In this work, kernels have been dehulled to avoid additional effects due to the natural resistance of the hull to fungal penetration [7] and to the presence of different classes of polyphenolic compounds in the rice husk [28]. In this way, the specific effect of the caryopsis coat pigmentation could be assessed without interference by the hull.

A preliminary test showed no inhibitory effect of the red pigment extract on growth of *F. graminearum*, *F. subglutinans* and *F. verticillioides*. Even at the highest concentration (8% of extract in PEG solution, corresponding to 13.9 mg of dry extract per mL of growth medium), the effect of red pigment extract on mycelial growth (expressed as mean percent inhibition in comparison with non-treated controls, ±SE) was null: 1 ± 3%, 1 ± 5%, and −2 ± 3% for the three aforementioned *Fusaria*, respectively. PEG alone did not show any effect at the concentrations used.

### 3.1. Germination and Growth of Caryopses Infected with E. nigrum

Dehulled caryopses, arranged in circle around the central disc with *E. nigrum*, were incubated at 10 °C to allow penetration of the fungus into the caryopses. The plates were then transferred to 30 °C for rice germination, and seedling growth was assessed after three days. Two independent experiments gave close results, and the data were therefore merged. The overall time of incubation at 10 °C was 14 days in one experiment and 17 days in the other. In both cases, the plates with the caryopses were transferred to 30 °C when some growth of the fungus was visible on the caryopses.

**Figure 1:**
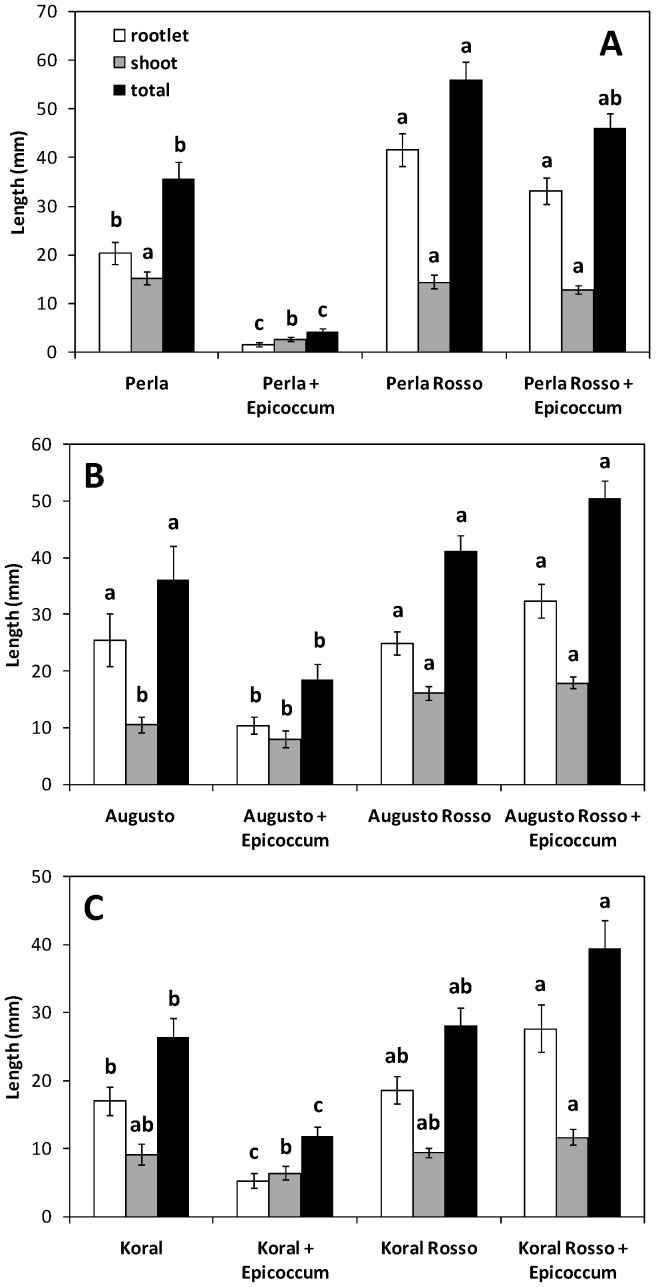
Seedling growth (rootlet length, shoot length, and their sum) in the presence or absence of *Epicoccum nigrum* for: (**A**) Perla and Perla Rosso; (**B**) Augusto and Augusto Rosso; (**C**) Koral and Koral Rosso. Dehulled caryopses were infected at 10 °C for 14–17 days and then kept at 30 °C for three days to allow rice germination and seedling growth. Values are means of five (controls) or eight (+*E. nigrum*) plates with 20 caryopses each (i.e., 100–160 caryopses) ± SE. ANOVA showed a significant effect (*p* < 0.05; *n* = 5–8 data for each mean) of both grain type (red/white), treatment (presence/absence of *E. nigrum* inoculum) and their interaction, for all white/red grained genotype pairs and every measure set (rootlet length, shoot length, and their sum) but for the treatment effect (on rootlet length, shoot length, and their sum) in both the Augusto and Augusto Rosso pair and Koral and Koral Rosso pair, as well as the interaction effect on shoot length in the Augusto and Augusto Rosso pair. For each white/red grained genotype pair and within each measure set (rootlet length, shoot length, and their sum), values with the same letter are not significantly different (*p* ≤ 0.05; Tukey’s test; *n* = 5–8 data for each mean).

The red blotch disease of rice grains can occur when rice plants lodge before harvest and the panicles come in contact with the soil, but damage incidence is seldom severe [5,16]. Figure 1 shows that for all the three white/red genotype pairs, the presence of *E. nigrum* strongly hindered seedling growth in the white-grained cultivar but not in the red-grained variety. In the Augusto/Augusto Rosso pair and, particularly, in the Koral/Koral Rosso pair, the red-grained counterpart seemed to actually improve seedling growth in the presence of *E. nigrum*. This effect could be due to the persistence (notwithstanding mild disinfection) of some contaminant pathogenic micro-organism on these caryopses, and an allopathic action of *E. nigrum* toward these pathogens. Limited growth of some contaminant micro-organisms was, in fact, occasionally apparent at the end of the test in the controls not treated with *E. nigrum* (see below). Thus, in the two above-mentioned comparisons, *E. nigrum* would seem to act as a potential pathogen for white-grained dehulled caryopses but as a potential biocontrol agent antagonising damping-off diseases for red-grained ones. Correspondingly, application of *E. nigrum* or its exudate protected cotton seedlings against *Pythium* damping-off and root-rot and enhanced their vigour and growth [29]. It has indeed been proposed that some non-pathogenic seed-associated microorganisms might suppress seed infections by pathogenic organisms [13,30]. In fact, *E. nigrum* produces antifungal compounds effective against pathogenic fungi [29,31,32,33,34,35]. As *E. nigrum* was able to grow at 10 °C while no growth of other contaminant micro-organisms was apparent at this temperature, when plates were transferred to 30 °C, antifungal compounds produced by *E. nigrum* could have blocked growth of other micro-organisms.

As mentioned above, untreated controls occasionally showed some fungal contamination. The degree of contamination was estimated by visual inspection of the plates before taking measurements of seedling growth. The apparent contamination was very low to undetectable (incidence ≤5%) for red-grained varieties and cultivar Augusto controls, low (≤10%) for cultivar Perla control, and moderate (≤20%) for cultivar Koral control. The growth of contaminant fungi was normally very low in the controls (that is, only tiny fluffy spots were usually observed). The degree of fungal contamination was not recorded for treated plates. A limited presence of contaminant microorganisms could also explain why the red-grained variety apparently showed a stronger seedling growth even in the controls not treated with *E. nigrum*: if the red coat provides a better protection to the caryopsis, this holds true for contaminant microorganisms as well. Thus, though grains were obtained from white-grained and red-grained plants that had been grown in the same environment, caryopses of the red-grained varieties should be better protected from occasional grain infections, and seedling growth can therefore have improved because seedlings were not affected by occasional microbial contaminations.

A further observation is that, in all the comparisons, the rootlet was more sensitive to the presence of *E. nigrum* (and of occasional contaminant microorganisms) than the shoot. It is indeed known that fungal attack is preferentially targeted to the radicle end of a germinating seed because of a gradient of nutrient exudates released from this vulnerable point on the grain [36].

Since our objective was to establish whether red caryopses are more resistant to infection, and all the red/white grained genotype pairs gave consistent results in this respect, we analyzed the data focused only on the caryopsis colour rather than on the individual genotypes. Besides, we distinguished whether the effect on seedling growth was due to lack of germination (previously considered as zero growth of both rootlet and shoot) or to reduced growth of the seedlings actually developed. Hence, mean seedling length (rootlet + shoot) was calculated only on germinated caryopses. A vigour index [24] was then calculated as the product of germination (%) times mean seedling length (rootlet + shoot, mm).

Table 1 shows that germination was significantly reduced by treatment with *E. nigrum* in white-grained genotypes but not in red-grained ones. All the three white-grained cultivars evidenced a reduction in seed germination of about 30–50% when the fungal inoculum was present with respect to the uninfected control. The total length of seedlings (excluding non-germinated caryopses) was reduced by fungal treatment as well. Accumulation of red pigment in the outer layers of the caryopsis is evidently a basic defence mechanism necessary to protect the caryopsis from microbial attack.

The outcome of fungus–seed interaction is also dependent upon the strength of the inoculum [37], which, in the presence of an *Epicoccum* mycelial disc, was very high. In addition, as the hull is a first mechanical barrier against micro-organisms challenging the intact grain [7], its removal has probably favoured infection by *E. nigrum*. Thus, in paddy conditions, that is, at low *Epicoccum* density, and in presence of intact grains (caryopses enclosed by the hull), this fungus is normally not pathogenic, only growing on dead tissues [5], although sometimes it becomes a weak pathogen [16]. Our more challenging experimental conditions were, however, useful to highlight the protective role of the red pigment in defying a microbial attack.

When germination and growth values were combined into the Vigour Index, the reduction of vigour by fungal treatment in white-grained genotypes but not in red-grained ones became even more evident (Table 1). It is well known that (white-grained) rice seeds infected by *E. nigrum* fail to germinate or produce weakened seedlings [5,16]. It has indeed been reported that seed germination and seedling growth are the stages of the life cycle at which seed-associated microbes potentially have their ultimate impact on viable seeds [30,36,37]. In fact, the fungal species present on healthy seeds in the soil usually do not reduce seed germination, but a number of them can be virulent to seedlings, therefore expressing their impacts as reduced seedling establishment without directly reducing seed viability and germination [30]. This has been suggested to be due to the rupture, at germination, of the protective structures enclosing the seed, which facilitates access for the pathogens [37,38].

### 3.2. Accumulation of T-2/HT-2 in Caryopses Infected with F. sporotrichioides

**Figure 2:**
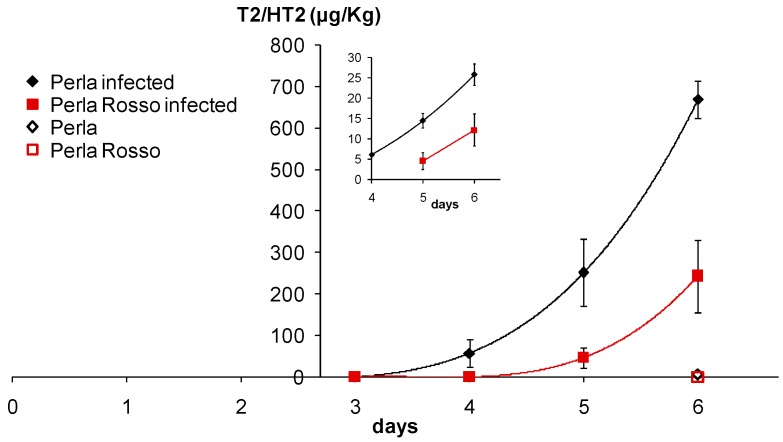
Accumulation of T-2/HT-2 toxins in dehulled caryopses of Perla (white caryopsis) and Perla Rosso (red caryopsis) incubated at 10 °C on agar in the presence of *Fusarium sporotrichioides* spores. Toxins were analyzed daily from three to six days after inoculation. Controls of dehulled caryopses without *F. sporotrichioides* spores were analyzed after six days only. ANOVA was therefore performed only on data of the last sampling day, showing a significant effect (*p* < 0.05; *n* = 4–7 data for each mean/point) of both grain type (red/white), fungal treatment and their interaction. The main plot shows the original data, whereas the inset displays data >0 (for infected plates) transformed to the square root (on the y-axis). This transformed dataset was analysed by including sampling day as a covariate in ANOVA, which thereby showed a significant effect (*p* < 0.001; *n* = 4–7 data for each mean/point) of both grain type (red/white) and sampling day.

Dehulled caryopses of Perla (white caryopsis) and Perla Rosso (red caryopsis) incubated at 10 °C on water agar either with or without spores of *F. sporotrichioides* were quantitatively analyzed for T-2/HT-2 toxins. Figure 2 shows that mycotoxin production was detected after four days of incubation in white caryopses, but after five days in red caryopses. This white/red grain genotype pair is of particular interest as it represents an ideal condition for testing the effect of pigmentation because of the same genetic background shared between the two genotypes, since Perla Rosso has been reported to be a natural red-grained mutant of the white-grained cultivar Perla [19].

The square root transformation was shown to nearly linearize the relationship between mycotoxin accumulation and sampling days after an initial lag time (inset in Figure 2). This means that mycotoxin production followed a power increase after an initial lag, and such lag was diverse for the two genotypes. Thus, to further analyze mycotoxin accumulation, data falling into the lag time, that is, before mycotoxin level increased above the baseline (i.e., values of zero or close to zero), were removed, including data for not infected controls. In this way, an evident linear trend was observed for each treated grain type (genotype) when values above the detection limit were considered (inset in Figure 2). The two trends were similar, indicating that penetration of *F. sporotrichioides* into red caryopses was hampered and delayed (by about one day) with respect to the white caryopses. The biological meaning of the lag can be interpreted as the time necessary for the fungus to access the nutritional reserves of the grain. The fact that mycotoxin accumulation followed a quadratic (or higher order) increase after the lag might suggest some multiplicative effect for mycotoxin production during fungal invasion. Indeed, in previous in planta experiments a close correlation between the amount of *F. sporotrichioides* DNA and the content of T-2/HT-2 mycotoxins has been found [39], and a reduction in mycotoxin contamination in grains could also limit fungal infection [11].

**Figure 3:**
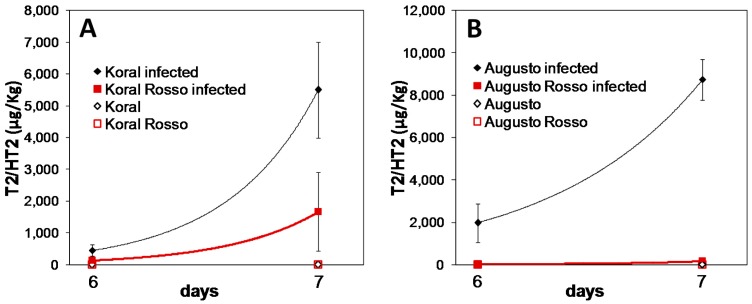
Accumulation of T-2/HT-2 toxins in dehulled caryopses of: (**A**) Koral (white caryopsis) and Koral Rosso (red caryopsis) and (**B**) Augusto (white caryopsis) and Augusto Rosso (red caryopsis), incubated at 10 °C on agar in the presence of *Fusarium sporotrichioides* spores. Toxins were analyzed after six and seven days from inoculation. Data for the two white/red grain genotype pairs were merged (thereby considering the grain colour type only, not the genotypes) and ANOVA showed a significant effect (*p* < 0.05; *n* = 4–6 data for each mean/point) of both grain type (red/white), fungal treatment (presence/absence of *F. sporotrichioides* inoculum) and sampling day, as well as of their two-term interactions and of the three-term interaction.

Since the difference between white and red caryopses was highly significant at six days of inoculation, the experiment was repeated comparing Koral with Koral Rosso (Figure 3A) and Augusto with Augusto Rosso (Figure 3B) at six and seven days of inoculation. In both instances, a lower level of toxins was observed in red caryopses with respect to white ones. The amount of mycotoxins in white caryopses showed a huge increase at seven days.

**Figure 4:**
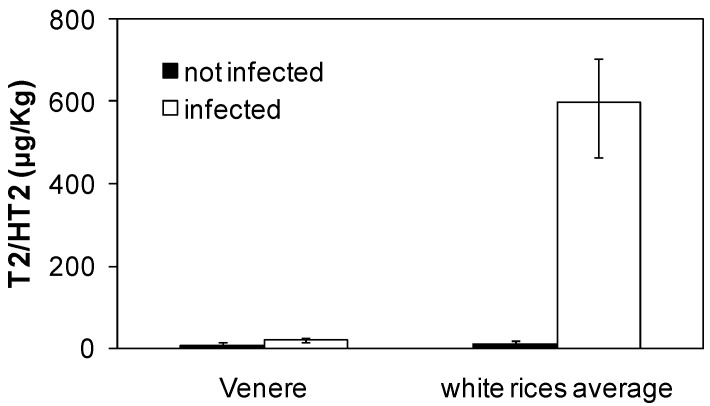
Accumulation of T-2/HT-2 toxins in dehulled caryopses of Venere (black caryopsis) with respect to the average of values obtained in previous tests for white-grained cultivars. As before, dehulled caryopses were incubated at 10 °C on agar either with or without *Fusarium sporotrichioides* spores. Toxins were analyzed after 6 days of inoculation. ANOVA showed a significant effect (*p* < 0.05; *n* = 3–10 data for each mean) of both grain type (black/white), treatment (presence/absence of *F. sporotrichioides* inoculum) and their interaction.

Beside red-grained cultivars, some purple/black grained rice genotypes are also known [2]. Cultivar Venere (with a black caryopsis) was used to ascertain whether even anthocyanins, which are responsible of the black colour of these kernels [2], can protect the caryopsis from micro-organisms. Figure 4 shows that this is indeed the case. Our results agree with several previous studies illustrating the favourable impact that flavonoids in general can exert against mycotoxins production [40].

Although even black caryopses showed reduced infection with respect to white ones and, therefore, anthocyanins have a protective effect against fungal infection analogous to that of PAs and PA-associated compounds, the red grain colour is ubiquitous among the wild ancestors of cultivated rice [18], which never display black caryopses. It may be hypothesised that since PAs undergo oxidative polymerization to insoluble phlobaphenes [3], in natural conditions they are less liable to leak out of the hull with respect to soluble anthocyanins. As no effect of red pigment extracts was observed on fungal growth on PDA, we argue that PAs, and/or the associated red pigment (phlobaphenes), are efficacious in protecting the caryopsis only if concentrated in a sort of barrier. In fact, in many species, PAs are deposited in the seed coat to constitute a pre-formed protective barrier [8]. This is a physical-chemical defence, necessarily arrayed on the exterior of the seed, that favours long-term persistence in the soil [38]. In red rice, a red-grained, weedy type of rice whose spikelets (caryopses covered by the hull) can persist dormant in the soil for one or more years, accumulation of PAs and PA-associated compounds seems to be an important defence strategy providing protection against biotic challenges [41]. Skadhauge et al. [10] also observed a moderate effect of extracts of PAs-accumulating barley testa layers on *Fusurium* growth, and concluded that the PAs and catechin in the testa layer of the mature barley grain are part of a passive resistance mechanism, acting as a physical barrier against pathogen attack.

## 4. Conclusions and Perspectives

Accumulation of (PA-derived) red pigment in the outer layers of the rice caryopsis ensures more protection against microbial attack, and, therefore, it enhances germinability and seedling vigour. Pigmentation thus appears to protect the seed from pathogens. The presence of this physical-chemical defence could then favour seed persistence in the soil, which would explain why wild *Oryza* species and weedy rices typically display a red caryopsis. Further research is however needed to clarify this aspect.

In addition, reduced infection by fungal contaminants can abate the mycotoxin content of the germinating grain. This might help to produce healthier sprouted rice for human consumption. Among the strategies for plant disease management, a broad resistance based on genetic factors already present in wild progenitors represents an appealing solution to control the development of fungal, particularly mycotoxigenic, species and then mycotoxin concentration in the (imbibed) cereal grain. Even the biological significance of mycotoxin production during fungal infection of grains deserves additional studies.

## Figures and Tables

**Table 1:** Rice germination and vigour after incubation at 10 °C for 14–17 days and then at 30 °C for three days. Values for white and red grain types are averages (± standard deviation among genotype means) of the three white-grained cultivars and of the three red-grained varieties, respectively. For every parameter (germination, mean seedling length, and vigour index), nested ANOVA showed a significant effect (*p* < 0.05; *n* = 15–24 data for cell mean) of both grain type (red/white), treatment (presence/absence of *Epicoccum nigrum* inoculum) and their interaction. For each parameter, values with the same letter are not significantly different (*p* ≤ 0.05; Tukey’s test; *n* = 15–24 data for cell mean).

Grain	Germination (%)		Mean Seedling Length (mm)		Vigour Index	
	Control	with *E. nigrum*	Control	with *E. nigrum*	Control	with *E. nigrum*
White	86.0 ± 7.9 a	52.9 ± 22.6 b	37.5 ± 4.9 a	20.2 ± 6.5 b	3262 ± 553 a	1140 ± 716 b
Red	96.0 ± 4.6 a	91.5 ± 4.7 a	43.0 ± 13.2 a	49.0 ± 4.4 a	4170 ± 1401 a	4515 ± 552 a
